# Community-Acquired Methicillin-Resistant Staphylococcus aureus Strain Positive for the Panton-Valentine Leucocidin Gene in a Middle-Aged Patient With Multiple Septic Pulmonary Emboli

**DOI:** 10.7759/cureus.56243

**Published:** 2024-03-15

**Authors:** Iori Moue, Masafumi Shimoda, Hiroyuki Kokutou, Tomoko Hanawa, Yoshiaki Tanaka

**Affiliations:** 1 Respiratory Disease Center, Fukujuji Hospital, Kiyose, JPN; 2 Department of General Medicine, Kyorin University Faculty of Medicine, Mitaka, JPN

**Keywords:** usa300, lip abscess, community-acquired, septic pulmonary emboli, mrsa (methicillin-resistant staphylococcus aureus), panton-valentine leukemia

## Abstract

A 59-year-old man suffered from fever and chest pain for three days following an accidental bite to a lip ulcer. His lower lip showed swelling and tenderness, and chest computed tomography showed multiple bilateral nodules. He was diagnosed with septic pulmonary embolism and a lip abscess, and blood, sputum, and lip abscess cultures confirmed the presence of methicillin-resistant *Staphylococcus aureus* (MRSA). Despite the initiation of vancomycin, he rapidly developed respiratory failure and septic shock, necessitating intubation and noradrenaline support. Gentamicin was added on the seventh day of admission due to an insufficient effect, and vancomycin was switched to linezolid on the 14th day of admission. However, his respiratory failure persisted as bilateral pneumothorax developed. Blood culture was negative on the 14th day after admission, but the patient died on the 15th day after admission. The MRSA isolate was tested for the presence of the Panton-Valentine leukocidin (PVL) gene in conjunction with the USA300 strain. The prevalence of community-acquired (CA)-MRSA in the USA300 clone is increasing but still low in Japan, and this type of infection is commonly observed in people of all ages; this case is the first instance reported in Japan of a middle-aged patient with septic pulmonary embolism. Given the anticipated global increase in CA-MRSA infection caused by the USA300 clone and the emergence of USA300 with altered pathogenicity, it may be crucial to suspect PVL-positive CA-MRSA infections even in middle-aged or elderly patients presenting with septic pulmonary embolism as community infections.

## Introduction

Panton-Valentine leukocidin (PVL) is a toxin that induces cell death through necrosis or apoptosis by releasing cytotoxic lysosomal granule contents from lysed neutrophils [1]. This toxin has been associated with community-acquired methicillin-resistant *Staphylococcus aureus* (CA-MRSA) infections worldwide and is a common cause of skin, soft tissue, and bloodstream infections in young healthy individuals [2,3]. In particular, septic pulmonary emboli and necrotizing pneumonia caused by PVL-positive CA-MRSA have been reported to have high mortality rates [4]. In the United States, the USA300 clone of CA-MRSA, which is positive for PVL and carries the type IV staphylococcal cassette chromosome *mec*, is a prevalent cause of CA-MRSA infections [3,5]. However, in Japan, the number of reported CA-MRSA cases caused by the USA300 clone has been relatively low, although there is an increasing expectation of its prevalence [6]. Most CA-MRSA isolates in Japan are PVL-negative, and there is limited information about the characteristics of CA-MRSA infections caused by the USA300 clone [3]. Herein, we present a rare case of CA-MRSA infection caused by the USA300 clone, which is positive for the PVL gene, in a middle-aged patient with multiple septic pulmonary emboli.

## Case presentation

A 59-year-old man with a five-day history of fever and chest pain lasting for three days visited our hospital. He had no significant medical or allergic history, although he was a current smoker (19.5 pack-years). He had not traveled abroad and had no recent contact with foreigners. Twelve days prior to admission, he developed a mouth ulcer. Subsequently, he experienced fever and chest pain following an accidental bite of the ulcer. He initially visited a local doctor, and chest computed tomography (CT) showed multiple bilateral nodules. Consequently, he was referred to our hospital.

**Figure 1:**
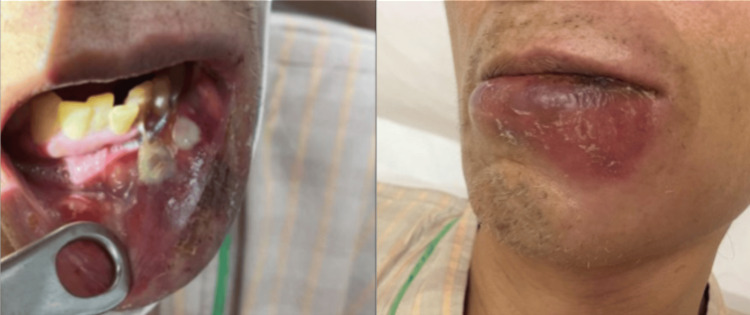
Swelling and tenderness in his lower lip, with inflammation expanding to the left side of his neck

**Figure 2:**
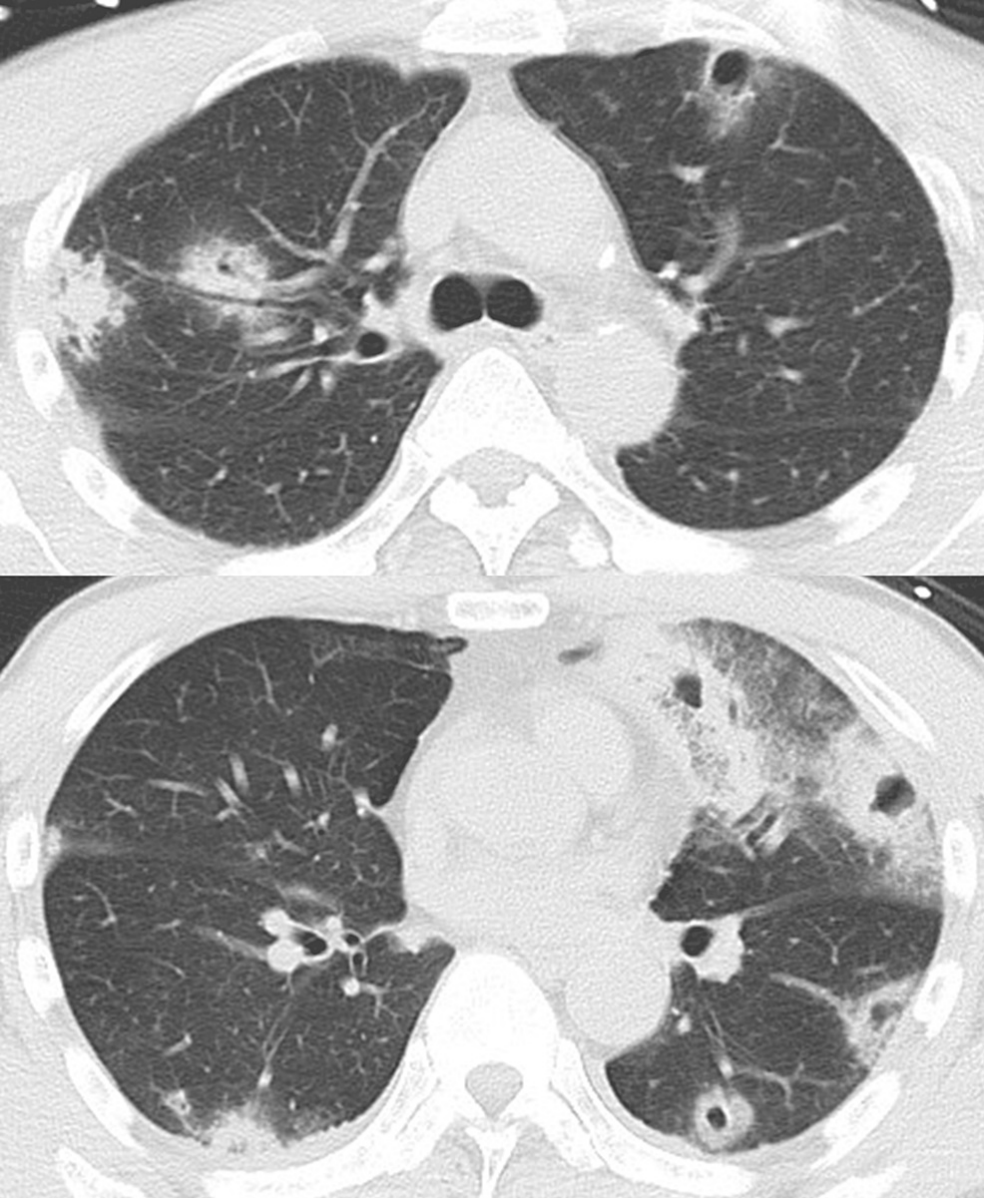
Chest computed tomography scans showed multiple bilateral nodules with cavities predominantly in the peripheral lung fields

The examination of vital signs indicated a low-grade fever of 37.0°C on admission, with no respiratory failure. Physical examination revealed swelling and tenderness in his lower lip, with inflammation expanding to the left side of his neck (Figure 1). The laboratory findings revealed a white blood cell count of 32,770 cells/µL, a C-reactive protein level of 28.78 mg/dL, and a procalcitonin level of 6.05 ng/mL (Table 1). Chest CT revealed multiple bilateral nodules with cavities predominantly in the peripheral lung fields (Figure 2).

**Table 1:** Laboratory findings upon admission

Hematology and biochemistry	Data
White blood cells (cells/μL) (normal range 3,300-8,600)	32,770
C-reactive protein (mg/dL) (normal range ≦0.14)	28.78
Procalcitonin (ng/mL) (normal range ≦0.055)	6.05

**Figure 3:**
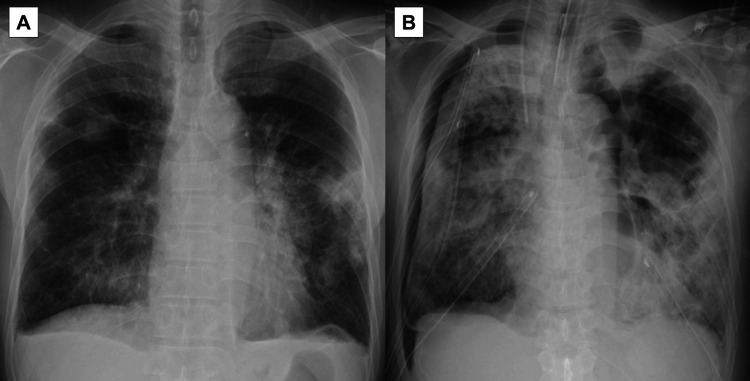
A) Chest radiography on the day of admission showed multiple nodular and infiltrating shadows in both lung fields. B) Chest radiography on the seventh day after admission showed bilateral pneumothorax

He was diagnosed with septic pulmonary emboli and a lip abscess, and treatment with tazobactam/piperacillin was initiated. However, blood culture on admission revealed methicillin-resistant *Staphylococcus epidermidis*, leading to the initiation of vancomycin treatment on the second day after admission. By the third day after admission, his chest radiography showed diffuse bilateral ground-glass opacities, and he rapidly developed respiratory failure and septic shock, necessitating intubation and noradrenaline support. Subsequent repeated blood cultures and sputum and lip abscess cultures on admission confirmed the presence of MRSA. Gentamicin was added on the seventh day after admission due to the repeated detection of MRSA in the 3rd round of blood culture, vancomycin was switched to linezolid on the 14th day after admission, and a blood culture returned negative on the 14th day of admission. However, his respiratory failure persisted as bilateral pneumothorax developed. Although chest drainage was performed, his lungs failed to re-expand (Figure 3). Finally, he died on the 15th day after admission. Ultimately, he was found not to be in an immunocompromised state.

**Figure 4:**
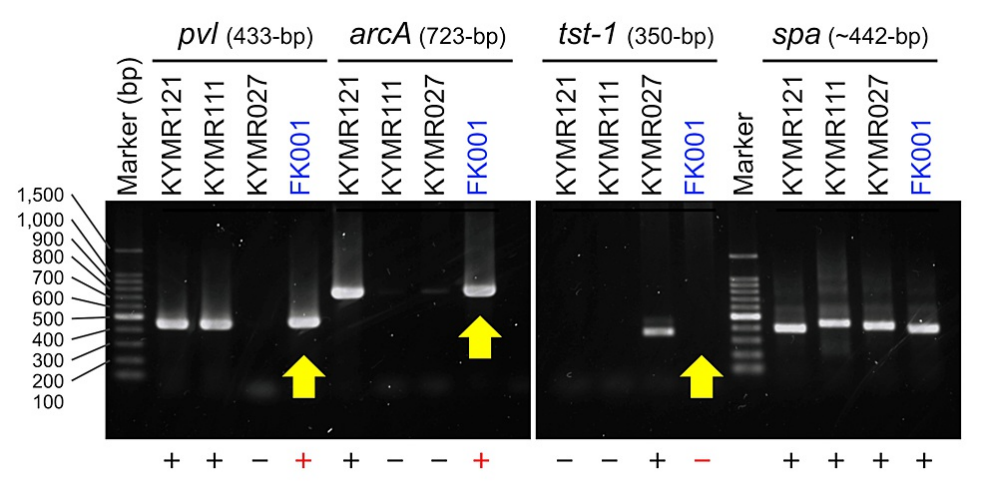
Genomic DNA was extracted from the clinical isolate of this patient, and from previous samples Genomic DNA was extracted from FK001, the clinical isolate of this patient, and from KYMR121, 111, and 027, which were previously isolated at Kyorin University Hospital, and the presence of the genes was assessed via PCR [7]. KYMR121 is positive for *pvl *(the Panton-Valentine leucocidin gene) and *arcA *(ACME-specific gene) because of the USA300 clone. KYMR111 is a PVL-positive strain but not a USA300 clone. KYMR027 is a *tst-1*-positive strain, and the toxic shock syndrome toxin-1 (*tst-1*) gene was tested. FK001 was positive for both *pvl* and *arcA* and negative for *tst-1* (yellow arrows), similar to KYMR121. The spa gene was used as a positive control for the reactions.

MRSA isolated from patient blood, sputum, and lip abscess cultures tested positive for the presence of the PVL gene and *arcA*, an arginine catabolic mobile element-specific gene, which is a feature of the USA300 clone (Figure 4).

## Discussion

This case represents a rare instance of septic pulmonary embolism with an intense course caused by the USA300 strain of CA-MRSA. Our patient died due to rapidly progressive destructive lung changes and secondary pneumothorax, which led to unimproved respiratory failure, despite the conversion of blood culture results to negative results. The condition of our patient rapidly deteriorated from no respiratory failure to requiring a ventilator within 24 hours. CA-MRSA strains are phylogenetically distinct from healthcare-acquired MRSA strains [5], and the virulence of PVL-positive CA-MRSA might be associated with a poor prognosis due to rapid disease progression [4]. PVL-positive CA-MRSA infections, including those caused by USA300 strains, were found in children and young adults and are found in people of all ages at present [3,6]. On the other hand, to our knowledge, only three cases of septic pulmonary embolism caused by the USA300 strain have been reported in Japan, and all of these patients were in their 20s or younger [8-10]. This case is the first reported instance in Japan of a middle-aged patient with septic pulmonary embolism caused by the USA300 strain of CA-MRSA.

Given that the anticipated emergence of the USA300 clone variant causes alterations in virulence and acquisition in healthcare settings [11,12], the number of cases among middle-aged and elderly individuals may increase in Japan. Consequently, even in regions such as Japan, where the incidence of PVL-positive organisms is increasing but still low [6], it may be crucial to suspect PVL-positive CA-MRSA infections in patients presenting with septic pulmonary embolism as community infections, which are primarily caused by *S. aureus *[13].

PVL is a toxin produced by *S. aureus* that targets leukocytes and macrophages, inducing cell death through necrosis and apoptosis [1,2]. Along with other properties specific to the USA300 clone, the toxin may affect clinical presentation, disease severity, and outcome, although further investigation is needed [1,2].

LZD and clindamycin (CLDM) have been recommended for treating PVL-producing bacteria due to their exotoxin-inhibitory effects [14]. In our case, VCM was switched to LZD; however, this change occurred the day before the patient’s death and after the lung had already undergone significant destruction. The initiation of LZD might have been too late to be effective. On the other hand, some reports of septic pulmonary embolism or necrotizing pneumonia caused by USA300 strains of CA-MRSA have shown rapid worsening and mortality, despite the early initiation of LZD and CLDM treatment [15-17]. In fact, two of three patients in whom LZD and CLDM were started within three days of admission died [15-17]. While recent reports suggest that the prognosis may not differ significantly from that of non-PVL-producing infections [2,18], these reports do not detail the treatments used, including LZD and CLDM, leaving it uncertain whether the exotoxin inhibitory effect plays a role in patient outcomes. Therefore, rapid progression can occur in patients with PVL-positive CA-MRSA infections, regardless of LZD and/or CLDM treatment.

There is no established treatment specifically for PVL-producing CA-MRSA [14]. In cases where septic pulmonary embolism progresses rapidly and causes lung destruction, as observed in our case, improving the condition may be challenging due to complications such as respiratory failure and pneumothorax, even if the infection itself can be controlled. Currently, it is advisable to administer LZD and CLDM to patients with septic pulmonary embolism, particularly to prevent lung destruction, considering the potential involvement of PVL-producing CA-MRSA [14]. However, this recommendation is based on limited evidence, and further research is necessary to substantiate these treatment strategies.

In this patient, methicillin-resistant *S. epidermidis* was detected via blood culture on the day of admission. However, subsequent repeated blood cultures revealed the presence of MRSA, and both sputum and lip abscess cultures taken on the day of admission also confirmed the presence of MRSA. Therefore, MRSA was determined to be the causative organism. The initial identification of methicillin-resistant *S. epidermidis* might be indicative of a mixed infection, although there is no definitive evidence to confirm this hypothesis.

## Conclusions

This case represents a rare instance of septic pulmonary embolism with an intense course caused by the USA300 strain of CA-MRSA. Although the prevalence of the CA-MRSA USA300 clone remains low in Japan, infections of this type are commonly seen across various age groups. It may be crucial to suspect PVL-positive CA-MRSA infections in patients presenting with septic pulmonary embolism as community infections, which are primarily caused by *S. aureus*.
